# Black-White Differences in Hearing Problems Among Older Americans: Findings From Two Large Representative Surveys

**DOI:** 10.1093/geroni/igaa057.1053

**Published:** 2020-12-16

**Authors:** Esme Fuller-Thomson, ZhiDi (Judy) Deng, Senyo Agbeyaka

**Affiliations:** University of Toronto, Toronto, Ontario, Canada

## Abstract

The purpose of this study is to investigate Black-White differences in hearing problems among older adults living in the United States. Secondary data analyses were conducted using the 2017 American Community Survey (ACS) with a replication analysis in the 2016 ACS. The ACS is an annual nationally representative survey of Americans living in community settings and institutions. The sample size of older Americans (age 65+) in 2017 was 467,789 Non-Hispanic Whites (NHW) and 45,105 Non-Hispanic Blacks (NHB). In the 2016 ACS, there were 459,692 NHW and 45,990 NHB respondents aged 65+. Measures of hearing problems, age, race/ethnicity, education level and household income were based on self-report. Data were weighted to adjust for non-response and differential selection probabilities. The prevalence of hearing problems was markedly higher among older NHW (15.4% in both waves) in comparison to NHB (9.0% in 2017; 9.4% in 2016; both p<.001). In the 2017 ACS, the age-sex adjusted odds of hearing loss were 69% higher for NHW compared to NHB, which increased to 91% higher odds when household income and education level were taken into account (OR=1.91; 95% CI=1.85, 1.97). Further analyses by 10 year age cohorts indicated comparable findings (fully adjusted ORs range from 1.89 to 1.98). Findings from the 2016 ACS were very similar (e.g., 65+ fully adjusted OR=1.81). NHW have a much higher prevalence and almost double the odds of hearing loss compared NHB. Future research should investigate if melanin plays an otoprotective role through enhancing the antioxidant capability of cochlea.

